# Ineffectiveness of lateral-wedge insoles on the improvement of pain and function for medial knee osteoarthritis: a meta-analysis of controlled randomized trials

**DOI:** 10.1007/s00402-018-3004-z

**Published:** 2018-07-20

**Authors:** Junfeng Zhang, Qin Wang, Cuiming Zhang

**Affiliations:** 1 Department of Health Statistics, Public Health of Shanxi Medical University, Taiyuan, 030001 Shanxi China; 2 Publishing house, Chinese Journal of Rheumatology, Taiyuan, 030001 Shanxi China; 3 Department of Ultrasound, The 2nd Hospital of Shanxi Medical University, Taiyuan, 030001 Shanxi China; 4 Department of Nephrology and Rheumatology, Shanghai Jiaotong University Affiliated Sixth People’s Hospital South Campus, Shanghai, China; 5 Department of Nephrology and Rheumatology, Fengxian Hospital Affiliated to Southern Medical University, Shanghai, 201400 China

**Keywords:** Lateral-wedge insoles, Medial knee osteoarthritis, Meta-analysis

## Abstract

**Objective:**

The study aimed to evaluate the role of lateral-wedge insoles in pain reduction and functional improvement among patients with medial knee osteoarthritis.

**Materials and methods:**

Three databases including Pubmed, Embase, and Web of science were searched from inception until October 2017 for studies investigating the role of lateral-wedge treatment in pain relief and functional recovery among patients with knee osteoarthritis. Eligible studies were pooled using fixed effect model or random-effects model based on Cochrane *Q* statistic and *I*^2^ test. Moreover, subgroup analysis stratified by research area was performed, and sensitivity analysis was further designed to evaluate the strength of the meta-analysis.

**Results:**

Ten studies with a total of 938 patients, of which 478 patients received lateral-wedge insoles and 460 patients were set as control, were included in the meta-analysis. The pooled statistics did not show significant improvement in knee pain (SMD = − 0.21, 95% CI − 0.50, 0.08; *P* = 0.16) and knee function (SMD = 0.22, 95% CI − 0.27, 0.70; *P* = 0.38) in lateral-wedge insoles treatment group compared with controls. However, subgroup analysis based on research area revealed a favorable outcome toward Asian patients who received lateral-wedge insoles in pain reduction when compared with control group. (SMD = − 0.88, 95% CI − 1.59, − 0.17; *P* = 0.02). No significant improvement was observed among patients in USA and other areas. Sensitivity analysis showed unchanged results when we omitted each study. No significant publication bias was observed among the included studies.

**Conclusion:**

Though for young Asian patients within normal BMI, to some extent, the lateral-wedge insoles seems to be helpful. However, there was no evidence to demonstrate the relationship between race and role of lateral-wedge insoles on pain reduction. All in all, based on current data, lateral-wedge insoles appear to be ineffective at attenuating knee pain and function improvement.

## Introduction

In the elderly population, osteoarthritis (OA) is a major chronic musculoskeletal pain leading to restricted movement and decreased physical activity [1]. Nonsurgical treatments such as exercise and orthotic insole were recognized as optimal management of OA [2, 3].

Clinical evidence put forward that 60–80% of the body weight is distributed to the medial compartment of the knee [4], which also suffers from the counter force from the ground during the distance phase with normal gait. Thus, the medial knee bear relatively large load. A review study suggested that the decreased load of the medial compartment in medial osteoarthritis patients with valgus brace or lateral wedge is beneficial for functional recovery and pain relief to some extent [5–9]. Laterally wedged insoles can change the stress point of knee–ankle–foot by elevating the lateral foot so as to reduce load of medial knee. Simply put, the medial edge was made thicker than the lateral by lateral-wedge insoles, which can help patients transfer loading from the medial to the lateral knee compartment [10]. In 1987, Sasaki introduced lateral-wedge insoles in the treatment for medial knee osteoarthritis so as to relieve the pressure in the knee joint by changing limb alignment [11]. Kerrigan et al. also have found that lateral-wedge insoles can effectively alleviate the symptoms of knee arthritis and improve the locomotive function of patients [12]; Some studies investigating knee pain following treatment suggested a larger amount of pain decreases when using wedged insoles [12–14]. Then, the insoles were recommended as a treatment for reducing the load on the medial knee compartment. As a consequence of this medial unloading, painful knee symptoms should be reduced. However, others studies suggested that patients achieved little pain reduction after using wedged insoles compared with a control treatment [15]. These inconsistent outcomes might cause by various factors, such as patients’ ethnicity, country of residence, treatment duration, and the physical distribution. Overall, there is no consensus regarding the efficacy of lateral-wedge insoles as a treatment for pain in medial knee OA.

A previous meta-analysis by Parkes et al. reported that lateral-wedge treatments was associated with significant pain reduction in OA patients when compared with controls [16], and patients with concomitant therapy alongside the treatment were also analyzed. However, to our knowledge, function improvement introduced by lateral-wedge treatments has not been meta-analyzed. In the present study, we aimed to assess the efficacy of lateral-wedge treatments as an independent therapy in knee pain reduction and function improvement for OA patients.

## Materials and methods

### Literature search

Randomized control studies investigating the role of lateral-wedge treatment in pain relief and function among patients with knee OA were searched from databases including Pubmed, Embase, and Web of science from database inception to October 2017. The applied search terms were “lateral-wedge insoles” and “osteoarthritis” in combination. Reference lists of the eligible studies were screened and relevant meta-analysis was hand searched to find additional publications.

### Study selection

Studies published in English were enrolled in the meta-analysis if they met the following criteria: (1) randomized controlled trials with a lateral-wedge treatment group and control (placebo or no treatment) group and (2) the enrolled patients were diagnosed with medial compartment knee osteoarthritis based on X-ray examination. Patients with concomitant therapy alongside the lateral-wedge treatment and the control were excluded. Moreover, the non-trail papers, including review, comment letters, and meeting reports, were excluded.

Two reviewers independently screened all the potential studies retrieved from the initial search according to the eligible criteria. Disagreements would be resolved by discussion until consensus was reached.

### Data collection and quality assessment

Data were extracted following a predefined information sheet. Briefly, the following characteristics were collected from each study: the first author, year of publication, population demographic characteristics, intervention, treatment duration, and treatment dosage.

Study quality was assessed using Cochrane Risk of Bias tool following key criteria: adequate sequence generation, allocation concealment, blinding, incomplete outcome data addressed, free of selective outcome reporting, and free of other bias. Overall risk of bias for each study was summarized as “low”, “unclear”, or “high” based on the risk of bias across each of the key criteria.

### Statistical analysis

The difference in mean changes of pain from baseline to follow up between patients receiving lateral-wedge therapy and control treatment was evaluated. The outcome was presented as standardized mean differences (SMD) with its 95% confidence intervention (95% CI). Heterogeneity among studies was examined using Cochrane *Q* statistic and *I*^2^ test. In meta-analysis, the usual way of assessing whether a set of single studies are homogeneous is by means of the *Q* test. The *Q* test is computed by summing the squared deviations of each study’s effect estimate from the overall effect estimate, weighting the contribution of each study by its inverse variance. Under the hypothesis of homogeneity among the effect sizes, the *Q* statistic follows a Chi square distribution with *k* – 1 degrees of freedom, k being the number of studies. However, the *Q* test only informs us about the presence vs the absence of heterogeneity, it does not report on the extent of such heterogeneity. The *I*^2^ index has been proposed to quantify the degree of heterogeneity in a meta-analysis. The *I*^2^ index measures the extent of true heterogeneity dividing the difference between the result of the *Q* test and its degrees of freedom (*k* – 1) by the *Q* value itself, and multiplied by 100. Therefore, it is similar to an intraclass correlation in cluster sampling [17]. The I^2^ index can be interpreted as the percentage of the total variability in a set of effect sizes due to true heterogeneity, that is, to between-studies variability. Higgins and Thompson [17] proposed a tentative classification of I^2^ values with the purpose of helping to interpret its magnitude. Thus, percentages of around 25% (*I*^2^ = 25), 50% (*I*^2^ = 50), and 75% (*I*^2^ = 75) indicates low, medium, and high heterogeneities, respectively.

In this study, *P* < 0.05 and/or *I*^2^ > 50% was defined as significant heterogeneity occurrence and the random-effects model was chosen to pool the effect size. Otherwise, the fixed effect model was used. Subgroup analysis stratified by researched area was performed to explore the source of heterogeneity. To further evaluate the strength of the meta-analysis, sensitive analysis was performed through omitting one study each time. Finally, the likelihood of publication bias was assessed by constructing funnel plots.

## Results

### Study selection

**Fig. 1:**
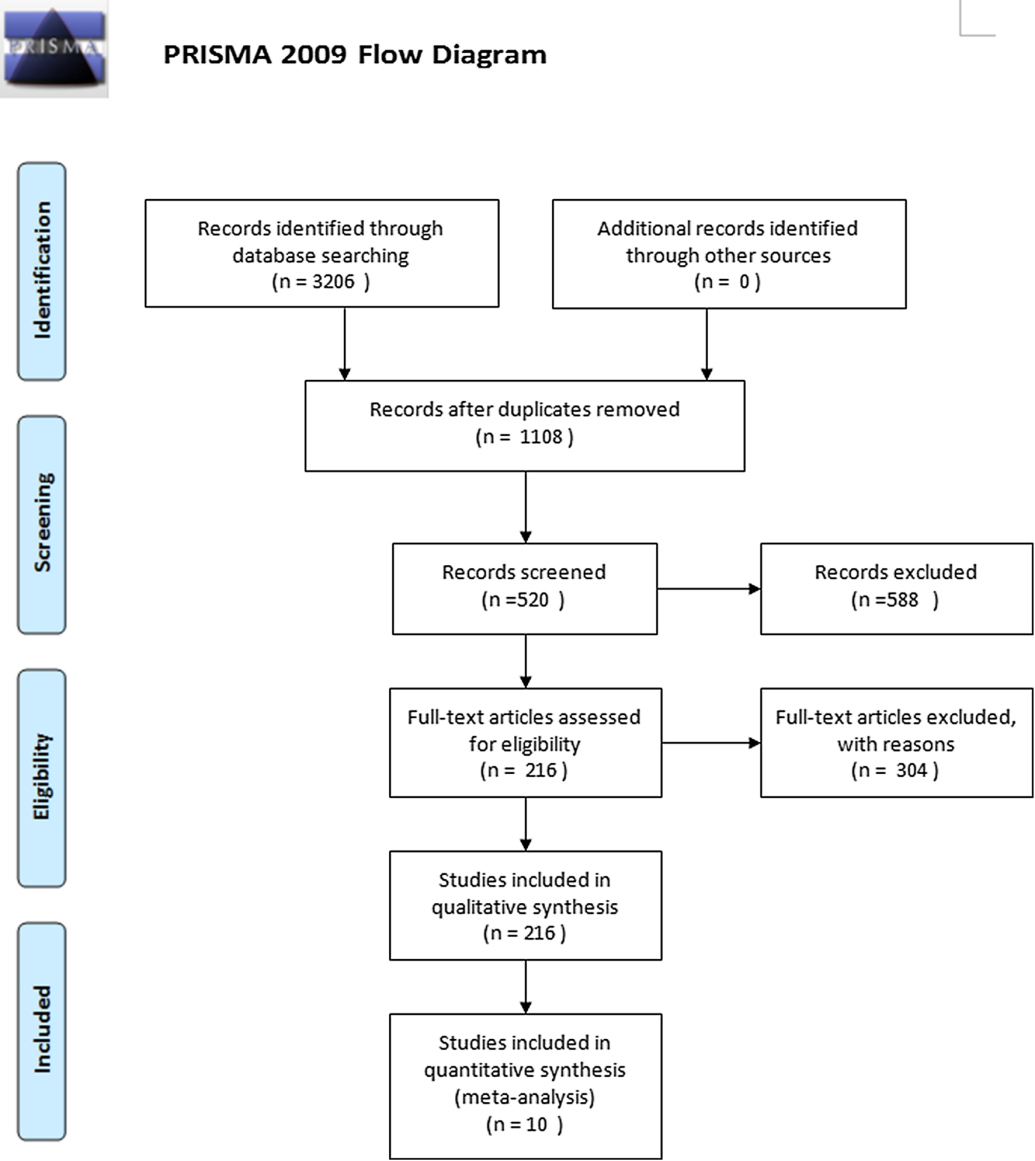
Flow diagram for the study selection

As shown in Fig. 1, 3206 articles were originally searched. After removing duplicate studies, 1108 abstracts were reviewed. Of these, 588 articles were excluded. Exclusion criteria were as follow: (1) subjects reported the use of assistive devices; (2) subjects with a history of any neurological, cardiopulmonary, or musculoskeletal condition that could affect ambulation; (3) clinical presentations of symptomatic lateral tibiofemoral and/or patellofemoral OA, such as peripatellar or lateral joint line pain; (4) subjects with foot conditions that could potentially be aggravated by a laterally wedged orthosis; (5) patients with concomitant therapy alongside the lateral wedge and control treatment; and (6) the non-trail papers, including review, comment letters, and meeting reports. The remaining 520 abstracts were further reviewed, and 304 articles were excluded including 165 non-trail studies and 139 non-knee OA studies. Then, 216 articles were fully reviewed. Among these articles, 23 with concomitant therapy, 85 articles did not report pain or function outcome, 39 articles were non-RCT design, 34 articles were duplicated populations, and data could not be extracted in 25 articles. Finally, ten articles were enrolled in the meta-analysis [18–27].

### Study characteristics

The characteristics of enrolled studies are summarized in Table 1. Of the ten trials, eight studies used a neutral insole as control [18–21, 23, 25–27], and two studies did not use any treatment [22, 24]. Treatment duration ranged from 6 weeks to 24 months. Except one study, the age of patients in seven studies was more than 60 years [18–23, 25].

**Table 1:** Characteristics of included studies

References	Country	Female, *n* (%)	Age	Body mass index	Intervention	Treatment dosage	Treatment duration	Outcome assessment
Baker et al. [18]	USA	Wedge to neutral: 24(53) Neutral to wedge: 27 (66)	Neutral to wedge: 67.8 (9.9) Wedge to neutral: 68.2 (8.7)	Wedge to neutral: 33.0(4.8) Neutral to wedge: 32.9(6.4)	T: 5° lateral-wedge insole; C: a neutral insole	Worn for 7–8 h/d	6 weeks	WOMAC
Bennell et al. [19]	Australia	Treatment: 62 (60) Control: 56 (58)	Treatment: 63.6 (8.1) Control: 65 (7.9)	Treatment: 28.1 (4.2) Control: 30.4(5.6)	Treatment group: LWI Control group: neutral insole	Wear the insoles full time in their shoes	12 months	WOMAC
Erhart-Hledik et al. [21]	USA	Treatment: 14 (41) Control: 15 (58)	Treatment: 61.4(9.2) Control: 62.1 (9.9)	Treatment: 27.6 (4.5) Control: 27.4(5.4)	Treatment group: bilateral neutrally wedged insoles Control group: Bilateral laterally elevated	Worn for 6.4–7.9 h/d	12 months	WOMAC
Pham et al. [23]	France	Treatment: 54 (66) Control: 61 (82)	Treatment: 64.0 (10.8) Control: 65.6 (9.9)	Treatment: 29.0 (5.6) Control: 28.5 (5.3)	Treatment group: LWI Control group: neutral insole	checked adherence at 6 months	24 months	WOMAC
Sattari et al. [24]	Iran	Treatment total: 20 Control: 20	Overall: 48	NA	Treatment group: LWI (approximately 6°) Control group: no treatment	Whenever patients wore shoes	9 months	Pain on walking (VAS)
Toda et al. [25]	Japan	Treatment: 38 (88) Control: 32 (84)	Treatment: 66.1 (8.6) Control: 64.6 (9.8)	Treatment: 24.7 (2.9) Control: 24.6 (3.1)	Treatment group: LWI Control group: neutral wedge (odor repellent)	5–10 h/day whenever wearing shoes	12 weeks	Lequesne Index
Hsieh et al. [22]	China (Taiwan)	Treatment: 38 (88) Control: 32 (84)	Overall: 61.0 (9.9)	Overall: 25.0 (4.7)	Treatment group: LWI Control group: no treatment	NA	6 months	The pain subscale score of the WOMAC; physical function subscale of the WOMAC assessment
Campos et al. [20]	Brazil	Treatment: 18 (62.1) Control: 19 (65.5)	Treatment: 65.2 (9.6) Control: 63.3 (7.5)	Treatment: 30.8 (6.1) Control: 30.3 (5.1)	a lateral-wedge insole with subtalar strapping (Group W), or a neutral insole with subtalar strapping (Group N–control)	worn for 5–10 h/day	24 weeks	VAS, the WOMAC and Lequesne questionnaires
Barrios et al. [26]	USA	Treatment: 19 (54.3); Control: 18 (60)	Treatment: 62.0 (7.4); Control: 62.8 (9.6)	Treatment: 34.2 (7.2) Control: 31.9 (6.9)	Treatment: LWI; Control: neutral wedge	NA	12 months	WOMAC
Maillefert et al. [27]	France	Treatment: 54 (65.9) Control: (82.4)	Treatment: 64 (10.8); Control: 65.6 (9.9)	Treatment: 29 (5.6); Control: 28.5 (5.3)	Treatment: LWI; Control: neutral wedge	NA	6 months	WOMAC

*WOMAC* Western Ontario and McMaster Universities Arthritis Index, *VAS* visual analog scale, *LWI* lateral-wedge insole, *N*/*A* not applicable, *T* treatment group (lateral-wedge insole), *C* control group (neutral insole), *W* group W (a lateral-wedge insole with subtalar strapping), *N* group N (a neutral insole with subtalar strapping)

Participants and assessors were both blinded in the study by Baker et al., and the blind design was not performed in other studies.

### Pain assessment of lateral-wedge insoles

**Fig. 2:**
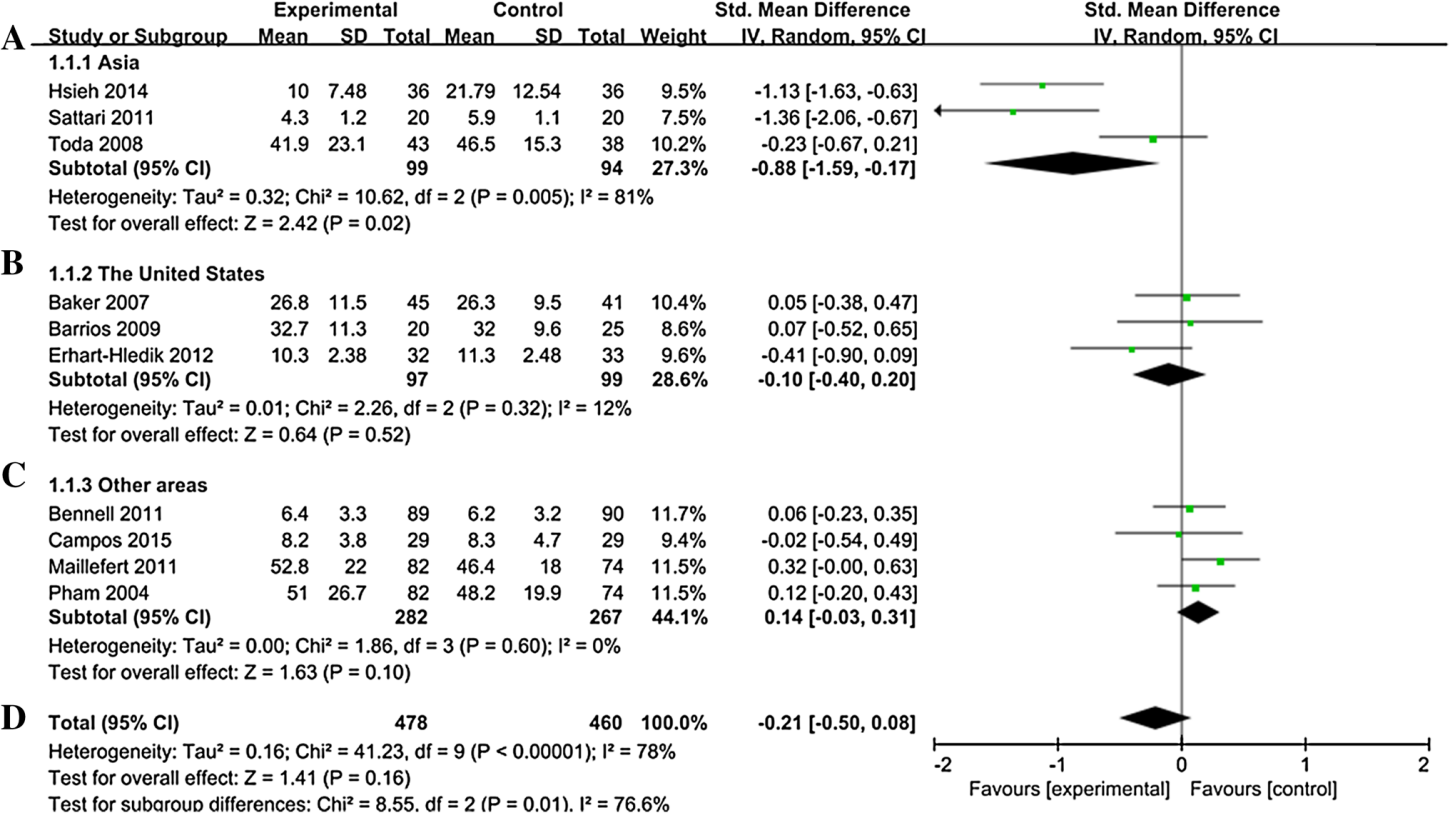
Forest Plot of heel wedge interventions on pain improvement. **a** Forest plot of heel wedge interventions on pain improvement among patients in Asia. **b** Forest plot of heel wedge interventions on pain improvement among patients in USA. **c** Forest plot of heel wedge interventions on pain improvement among patients in other areas. **d** Forest plot of heel wedge interventions on pain improvement

The effect of lateral-wedge insole on pain reduction was evaluated in all enrolled studies including 478 patients who received lateral-wedge insoles vs 460 patients who did not have the intervention [18–27]. As shown in Fig. 2d, significant heterogeneity was observed among individual studies evaluating knee pain (*I*^2^ = 78%, *P* < 0.001). Thus, a randomized effects model was used to pool data. No significant difference was observed between patients treated with lateral wedges and controls (SMD = − 0.21, 95% CI − 0.50, 0.08; *P* = 0.16). When studies were stratified according to study area (Fig. 2a–c), heterogeneity among studies researched in USA (*I*^2^ = 12%, *P* = 0.32) and other areas (*I*^2^ = 0%, *P* = 0.60) were significantly reduced. Pooled statistics revealed no beneficial effect on lateral-wedge insoles treatment as compared with controls both in USA (SMD = − 0.10, 95% CI − 0.38, 0.18; *P* = 0.50) and other areas (SMD = − 0.14, 95% CI − 0.03, 0.31; *P* = 0.10).

**Fig. 3:**
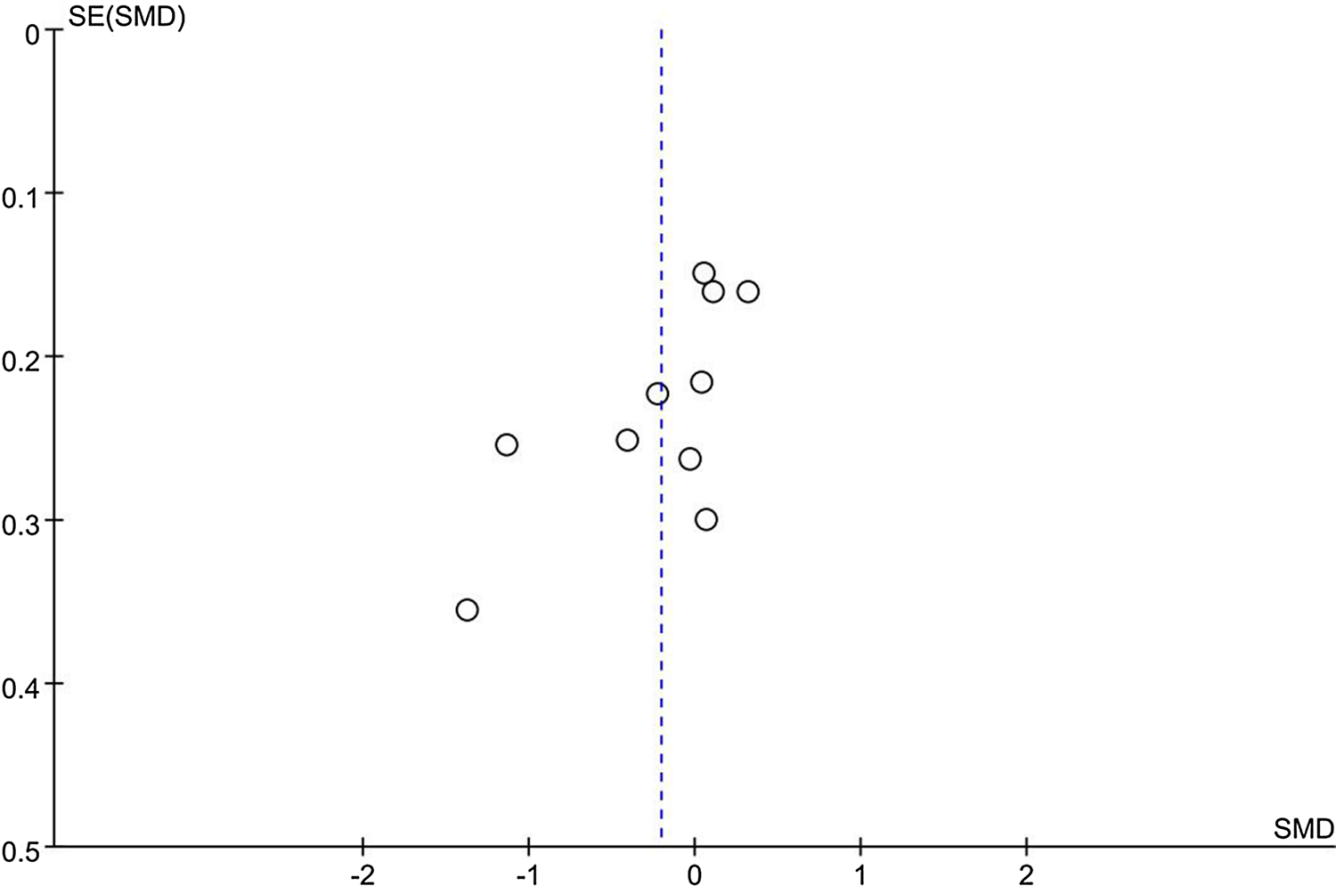
Funnel plots for heel wedge interventions on pain improvement

Notably, subgroup analysis on studies conducted with Asian population showed significant pain reduction in patients after lateral-wedge insoles treatment compared with controls (SMD = − 0.88, 95% CI − 1.59, -− 0.17; *P* = 0.02). Significant heterogeneity was observed (*I*^2^ = 81%, *P* = 0.005) among studies. However, although significant heterogeneity was observed in the meta-analysis, when we omitted each study in the sensitivity analysis, the results did not change, suggesting the relative stable strength of the results. Moreover, as shown in Fig. 3, the handstand symmetrical shape funnel plot suggested that there was no significant publication bias in the meta-analysis.

### Function evaluation of lateral-wedge insoles

**Fig. 4:**
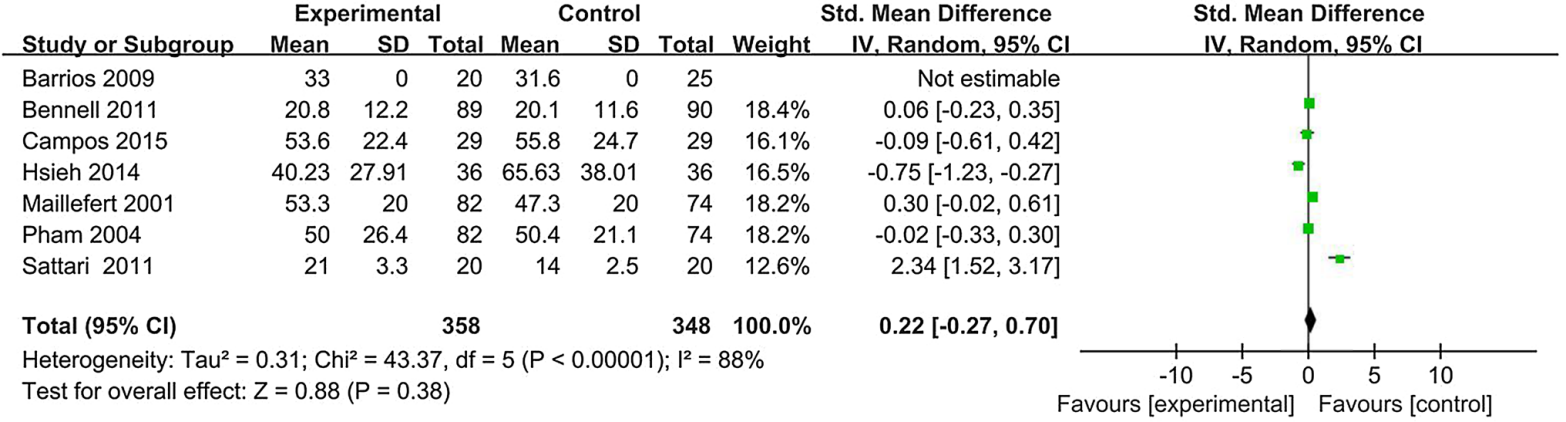
Forest plot of heel wedge interventions on function improvement

**Fig. 5:**
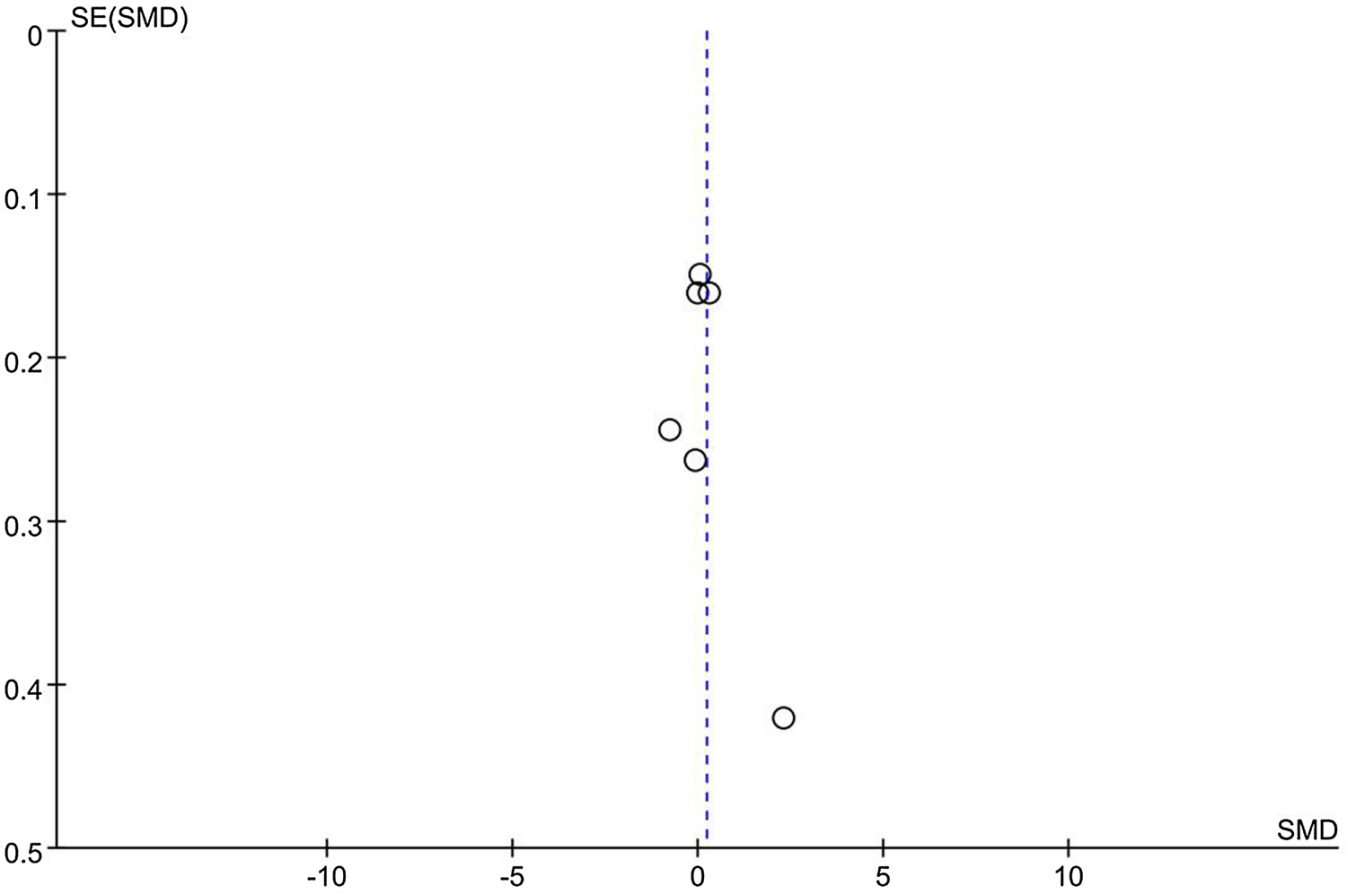
Funnel plots for heel wedge interventions on function improvement

Figure 4 shows that seven studies including 358 patients using lateral-wedge insoles and 348 controls were enrolled in the analysis of knee function recovery [19, 20, 22–24, 26, 27]. No significant difference was found in the function score between lateral-wedge insoles treatment group and controls (SMD = 0.22, 95% CI − 0.27, 0.70; *P* = 0.38). Significant heterogeneity was also observed among studies (*I*^2^ = 88%, *P* < 0.001). In sensitivity analysis, the results stayed unchanged when we omitted each study. Figure 5 shows that the funnel plot was as handstand symmetrical shape, suggesting no significant publication bias in the meta-analysis.

## Discussion

Based on the pooled results of the current meta-analysis, lateral-wedge insoles as an independent treatment appeared to be ineffective in attenuating knee pain and function improvement for knee OA patients. Subgroup analysis demonstrated that lateral-wedge insoles had a beneficial effect in Asian population. However, the existence of significant heterogeneity among the included studies might weaken the statistical power of the conclusion.

Many previous evidence have been established and suggested that lateral-wedged insoles could result in physical functioning improvement in patients with knee OA, such as increasing walking speed and reducing knee adduction angular [28, 29]. Studies suggested that the relevant biomechanical effects, including medial knee-joint-space load, adduction moment, and varus malalignment, were reduced after lateral-wedge insoles treatment [4, 12]. However, other studies reported that wedges might influence the normal foot and biomechanics associated with ankle and foot, which might aggravate the OA symptoms [30, 31]. Our study failed to find significant function improvement in medial knee OA patients who received lateral-wedged insoles treatment. The biomechanical changes in patients were complex when using the insoles. Moreover, other concomitant variables, including foot position, the severity of knee OA, and walking habits, might influence the function assessment [32]. In addition, medial knee stability is an important characteristic of overall knee health and the accurate measurement of medial knee gap width, which is critical to in properly diagnosing and evaluating knee condition, may also be a affecting variable [33]. Therefore, a further multi-centre randomized controlled design studies after adjusting the complex factors were needed to verify the conclusion.

It has been proved that the biomechanics of insoles were reduced in lateral knee external movements during gait analyses [34, 35]. Arnold and his colleagues put forward that lateral-wedge insoles could cause small reductions in the first and second peaks [36], which implicated in both the development of knee pain and radiographic progression of medial knee OA in older adults [37, 38]. However, when we examined their effect on pain and function improvement, data from our meta-analysis demonstrated that lateral-wedge insole treatment was of benefit when treating medial knee OA for Asian. It seems that lateral-wedge insoles were ineffective at pain reduction among other races patients.

Some studies suggested that the treatment effect of lateral-wedge insoles for medial knee OA patients with younger age [39] and lower body weight [40] was more likely to be satisfactory. In the three articles enrolled Asian patients [24, 41, 42], the average age was 48, 64.3, and 61.0, respectively. By comparison with that of patients in the USA and other areas, the Asian patients seems to be younger. Body mass index (BMI) is used to categorize a person by weight. BMI can simply reflect systemic overweight and obesity. As shown in Table 1, BMI of Asian patients were significant lower than that of Uniter states and other countries. Although there is no exact BMI in one study (Sattari), it should be notice that the study has excluded patients with BMI greater than 30. Overall, a proper deduction showed that the BMI of Asian patients is more likely in normal range 18.5–24.9 according to WHO standard. However, BMI of American patients and other countries are obviously higher than 25, most of them very close to 30, and even up to 33. By and large, the average age and body weight of Asian patients are more positive for the therapeutic effects of lateral-wedge insoles. Until now, there was no evidence to demonstrate the relationship between race and role of lateral-wedge insoles on pain reduction. However, we recognized that different constitutions of body and living habits might be reasons for the different conclusions. Therefore, additional studies stratified by living habits and other factors affecting control balance were required.

Several meta-analyses on the evaluation of the role of lateral-wedge insoles in pain reduction among patients with medial knee OA have been conducted. Until now, we only have found four meta-analyses [14, 43–45] on this topic. Three articles [14, 43–45] concluded that there were no major beneficial effects with the use of lateral wedges among patients with medial knee OA, which was in line with the conclusion of our study. Only one article [44] suggested that lateral-wedged insoles were in attempts to reduce osteoarthritic pain of biomechanical origin. Only one article [14] assessed the statistical heterogeneity using Chi-squared test and Cochran’s *Q* statistic. Thus, whether lateral-wedge insoles is efficient in the treatment for medial knee OA remains controversial.

Several limitations should be noted in the meta-analysis. First, significant heterogeneity existed among studies. Despite performing sensitivity analysis to diminish the impact of heterogeneity, the effect of heterogeneity still cannot be eliminated completely. Subgroup analysis reminded us that race might be one reason causing heterogeneity. Moreover, based on characteristics of the enrolled studies, the following potential factors should also be taken into consideration: the quality of the trial and the study design, the type of treatment used in control group (no treatment and neutral insole), and the treatment duration. Second, recovery time more than 12 months, various treatment duration and short follow-up time in the meta-analysis might affect the compactness of the study.

Though for young Asian patients within normal BMI, to some extent, the lateral-wedge insoles seems to be helpful. However, there was no evidence to demonstrate the relationship between race and role of lateral-wedge insoles on pain reduction. In conclusion, current data demonstrate that lateral-wedge insoles appear to be ineffective at attenuating knee pain and function improvement.
